# Long-Term Cutaneous Hyperpigmentation During Adjuvant Osimertinib Therapy in a Resected Stage IIB EGFR L858R-Mutated Lung Adenocarcinoma in an Older Adult: A Rare Case Report with Two-Year Follow-Up and Literature Review

**DOI:** 10.3390/curroncol33090536

**Published:** 2026-09-03

**Authors:** Marclesson Santos Alves, Juliana Palácio de Queiroz Ventura Barros, Danielle Calheiros Campelo Maia, Igor Santos Costa, Ormando Rodrigues Campos Junior, Howard Lopes Ribeiro Junior

**Affiliations:** 1 Pronutrir Oncology Clinic, Fortaleza 60810-180, CE, Brazil; alves.marclesson@gmail.com (M.S.A.); juliana.palacio@pronutrir.com.br (J.P.d.Q.V.B.); oncologia.danielle@gmail.com (D.C.C.M.); ormandojr@icloud.com (O.R.C.J.); 2 Argos Patologia, Fortaleza 60175-047, CE, Brazil; igorscosta@gmail.com; 3 Post-Graduate Program in Pathology, Post-Graduate Program in Translational Medicine, Center for Research and Drug Development (NPDM), Federal University of Ceará, Fortaleza 60430-275, CE, Brazil

**Keywords:** non-small cell lung cancer, EGFR mutation, L858R, osimertinib, cutaneous hyperpigmentation, violaceous rash, dermatologic toxicity, adjuvant therapy, case report, EGFR tyrosine kinase inhibitor

## Abstract

The use of targeted therapies has improved outcomes in patients with resected epidermal growth factor receptor-mutated non-small cell lung cancer, but uncommon and persistent dermatologic toxicities remain poorly characterized. We report a 71-year-old woman with resected lung adenocarcinoma harboring an epidermal growth factor receptor exon 21 L858R mutation. Following right upper lobectomy and systematic mediastinal lymph node dissection, pathological staging was pT2aN1M0 (stage IIB). She received four cycles of adjuvant cisplatin plus pemetrexed followed by planned three-year adjuvant Osimertinib. During Osimertinib treatment, she developed a persistent violaceous rash accompanied by progressive cutaneous hyperpigmentation. Osimertinib was temporarily interrupted, and dermatologic evaluation was performed, resulting in partial clinical improvement. Hyperpigmentation, however, persisted during follow-up. Other adverse events included grade 1 diarrhea, transient arthralgia, anorexia, and weight loss. Osimertinib was subsequently resumed and maintained. Nearly two years after surgery, the patient remains free of disease recurrence, with stable pigmentary skin changes. This case highlights an uncommon and prolonged dermatologic manifestation associated with Osimertinib and emphasizes the importance of recognizing atypical cutaneous toxicity to facilitate appropriate management and to facilitate appropriate dermatologic evaluation and individualized management of treatment-related toxicity.

## Simple Summary

Lung cancer treatment has improved with medicines that specifically target changes in tumor cells. Osimertinib is an important treatment for some patients whose lung cancer has an epidermal growth factor receptor mutation, including after surgery. Although skin problems are among its known side effects, unusual and persistent changes in skin color are less well-described. We report the case of a woman who developed a persistent purple-colored rash followed by marked darkening of the skin while receiving Osimertinib after surgery and chemotherapy for lung cancer. The skin changes improved partially after temporarily stopping treatment but remained stable after Osimertinib was restarted. The patient continued treatment without cancer recurrence. This case highlights the importance of recognizing uncommon skin reactions so that patients can receive appropriate dermatologic evaluation and, when clinically appropriate, continue their prescribed cancer treatment rather than stopping it unnecessarily.

## 1. Background

Lung cancer remains the leading cause of cancer-related death worldwide and accounts for approximately one in five cancer deaths [1]. Non-small cell lung cancer (NSCLC) accounts for nearly 85% of all lung cancers and is often diagnosed at an advanced stage, contributing to its poor prognosis [2]. Molecular characterization has become an integral part of NSCLC management, particularly in tumors harboring actionable driver mutations. Among these, activating alterations in the epidermal growth factor receptor (EGFR) are particularly relevant in lung adenocarcinoma and provide an established target for EGFR tyrosine kinase inhibitors (TKIs) [2,3,4]. Exon 19 deletions and exon 21 L858R substitutions comprise approximately 85–90% of EGFR-mutated tumors and are associated with sensitivity to EGFR-TKIs [5].

Osimertinib is a third-generation, irreversible EGFR-TKI that selectively inhibits sensitizing EGFR mutations and the T790M resistance mutation while sparing wild-type EGFR [6]. Initially developed for acquired resistance to earlier-generation EGFR-TKIs, it subsequently demonstrated superior efficacy in the first-line setting and became the preferred EGFR-targeted therapy for advanced EGFR-mutated NSCLC [7]. In the phase III FLAURA trial, osimertinib significantly prolonged progression-free survival compared with gefitinib or erlotinib, with a median progression-free survival of 18.9 months versus 10.2 months, respectively (hazard ratio [HR], 0.46; *p* < 0.001). In the final overall survival analysis, median overall survival was 38.6 months with osimertinib versus 31.8 months with comparator EGFR-TKIs (HR for death, 0.80; *p* = 0.046) [8,9].

More recently, the phase III ADAURA trial demonstrated a substantial disease-free survival benefit among patients with completely resected stage IB–IIIA EGFR-mutated NSCLC treated with adjuvant osimertinib after surgery, with or without platinum-based chemotherapy. At 24 months, 90% of patients with stage II–IIIA disease receiving osimertinib remained alive and disease-free compared with 44% of those receiving placebo (HR for disease recurrence or death, 0.17; *p* < 0.001) [9]. With longer follow-up, the final ADAURA analysis demonstrated a 5-year overall survival of 85% versus 73% among patients with stage II–IIIA disease (HR for death, 0.49; *p* < 0.001), and 88% versus 78% in the overall stage IB–IIIA population [10]. These findings established adjuvant Osimertinib as the standard of care for resected EGFR-mutated NSCLC [10]. Although generally well tolerated, Osimertinib commonly causes mild dermatologic, gastrointestinal, and nail toxicities, including acneiform rash, xerosis, pruritus, and paronychia [11,12]. However, atypical and persistent pigmentary alterations remain poorly characterized, particularly in the adjuvant setting.

Herein, we report a 71-year-old woman with completely resected stage IIB EGFR L858R-mutated lung adenocarcinoma who developed a persistent violaceous eruption followed by long-lasting cutaneous hyperpigmentation during adjuvant osimertinib therapy. The case is notable for the combination of a drug-induced interface dermatitis pattern on skin biopsy, progressive evolution from an inflammatory violaceous eruption to persistent post-inflammatory hyperpigmentation, and the ability to control the inflammatory component while maintaining osimertinib therapy. This case report was prepared in accordance with the CARE (CAse REport) guidelines [13].

## 2. Case Presentation

### 2.1. Patient Information and Clinical Findings

A 71-year-old Brazilian woman was referred to the Thoracic Oncology Department for evaluation of a newly diagnosed pulmonary malignancy after developing a persistent nonproductive cough for two months. She denied hemoptysis, chest pain, constitutional symptoms, or significant dyspnea. Her functional status was preserved, with mild exertional limitation (modified Medical Research Council (mMRC) grade 1). Baseline assessment showed obesity (body mass index (BMI), 31 kg/m^2^), with no evidence of malnutrition.

Her medical history included differentiated thyroid carcinoma, treated with total thyroidectomy and radioactive iodine therapy approximately ten years earlier, with no evidence of recurrence since then. She also had hypertension, type 2 diabetes mellitus, and dyslipidemia and was taking losartan, lercanidipine, evogliptin, and levothyroxine. She had a remote smoking history of approximately two pack-years and had stopped smoking more than three decades earlier. Her family history was notable for breast cancer in her mother and paternal aunt, gastric and prostate cancer in her father, and possible non-melanoma skin cancer in her paternal grandmother. At presentation, physical examination was unremarkable, and her ECOG performance status was 0, with preserved cardiopulmonary function. Pulmonary function testing showed adequate respiratory reserve for curative-intent surgical resection.

### 2.2. Diagnostic Assessment

**Figure 1:**
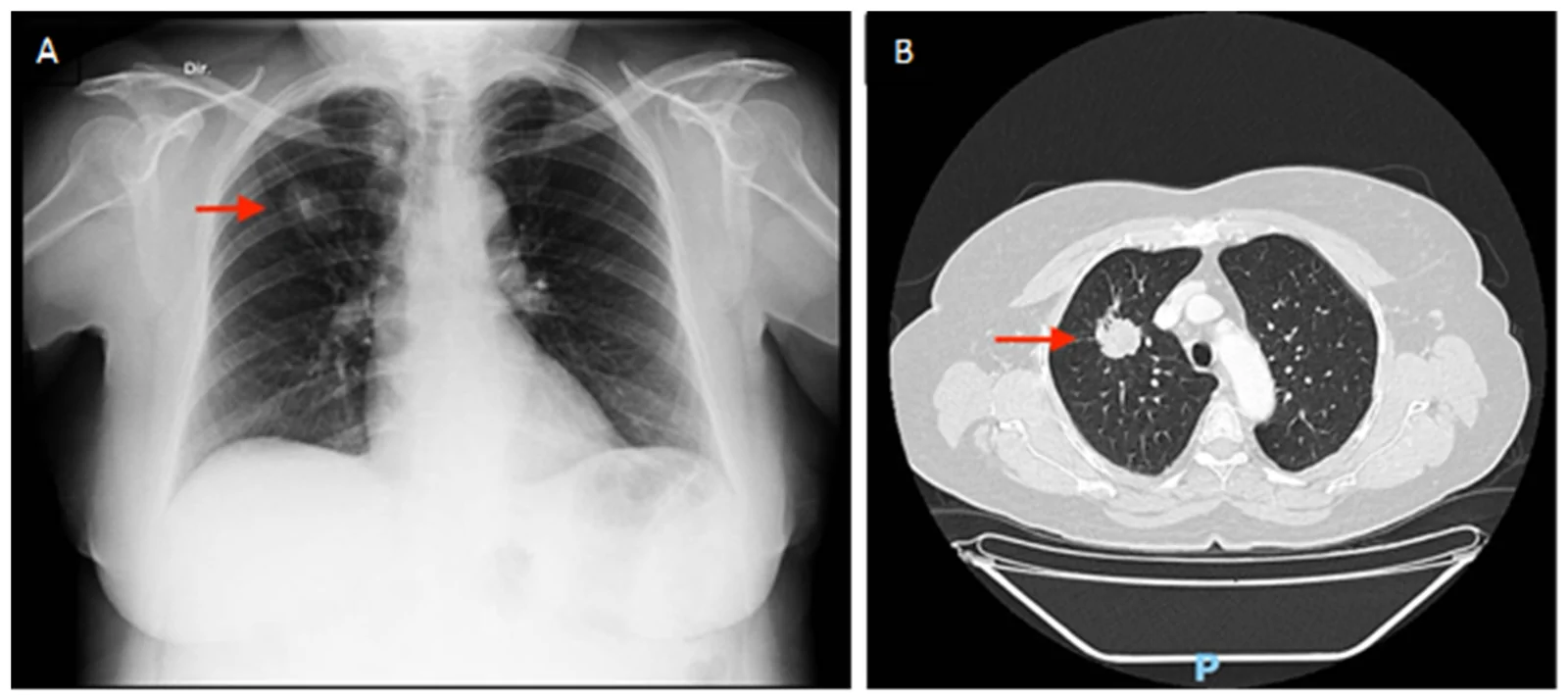
Initial chest imaging demonstrating the right upper lobe pulmonary nodule. Red arrows indicate the pulmonary lesion. (**A**) Chest radiograph showing the pulmonary lesion in the right upper lung field. (**B**) Axial chest computed tomography demonstrating a lobulated 2.9 cm solid nodule in the right upper lobe.

**Figure 2:**
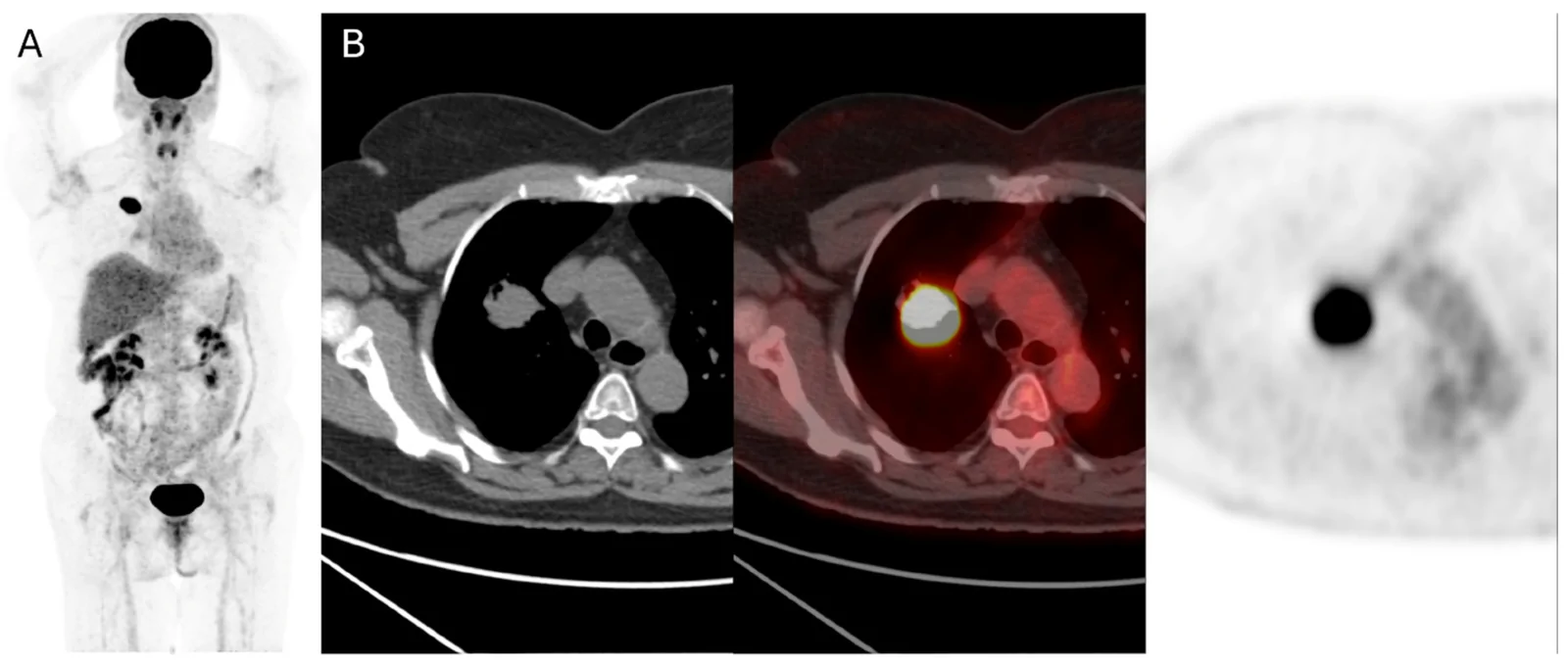
Baseline 18F-FDG PET/CT demonstrating intense metabolic activity in the right upper lobe lesion. (**A**) Whole-body maximum intensity projection (MIP) demonstrating an isolated hypermetabolic lesion in the right upper lung field, with no evidence of extrathoracic 18F-FDG-avid disease. (**B**) Axial CT, fused PET/CT, and PET images confirming a well-defined right upper lobe pulmonary nodule exhibiting intense 18F-FDG uptake, consistent with a metabolically active primary lung tumor.

Chest radiography (Figure 1A) and computed tomography (Figure 1B) revealed a lobulated 2.9 cm solid nodule in the right upper lobe with small pericardiophrenic lymph nodes. Subsequent ^18^F-fluorodeoxyglucose (FDG) positron emission tomography/computed tomography (PET/CT) demonstrated intense metabolic activity within the lesion (SUVmax 16.8), without evidence of distant metastatic disease (Figure 2). Brain magnetic resonance imaging showed no intracranial metastases. CT-guided percutaneous biopsy confirmed pulmonary adenocarcinoma.

### 2.3. Molecular Diagnostic Assessment

Initial molecular testing was performed using the ARGEN 50 next-generation sequencing (NGS) panel on the diagnostic lung tumor specimen. Histopathological review demonstrated invasive pulmonary adenocarcinoma with a predominantly acinar growth pattern. Molecular analysis identified an activating EGFR exon 21 L858R mutation [c.2573T>G; p.(L858R)] with a missense variant allele frequency of 9.72%. No pathogenic alterations were detected in ALK, BRAF, ERBB2, KRAS, MET, RET, ROS1, NTRK1, NTRK2, or NTRK3 (Table 1). Comprehensive genomic profiling with FoundationOne CDx confirmed the EGFR L858R driver mutation and identified additional genomic alterations, including CD274 (PD-L1) exon 7 rearrangement, APC Q1549, MTAP loss, CDKN2A loss, CDKN2B loss, RBM10 E380, and RNF43 S607L.

A subclonal DNMT3A S770L variant was also detected and was considered potentially related to clonal hematopoiesis (Table 1). Biomarker analysis demonstrated a microsatellite-stable (MSS) phenotype and a tumor mutational burden (TMB) of 4 mutations/Mb. No additional actionable alterations were identified in ALK, BRAF, ERBB2, KRAS, MET, RET, or ROS1, supporting EGFR-directed therapy as the principal molecularly guided therapeutic strategy.

### 2.4. Histopathological Assessment

Following multidisciplinary evaluation, the patient underwent right upper lobe lobectomy with systematic mediastinal lymph node dissection (Month 0). Histopathological examination revealed a 3.8 cm moderately differentiated invasive adenocarcinoma (grade 2). No evidence of spread through air spaces (STAS), lymphovascular invasion, or visceral pleural invasion was observed. Metastatic involvement was identified in one of sixteen resected lymph nodes. The final pathological stage was pT2aN1M0, corresponding to stage IIB disease according to the American Joint Committee on Cancer (AJCC) 8th edition staging system. Immunohistochemical evaluation using the VENTANA PD-L1 (SP263) assay revealed PD-L1 expression in 20% of tumor cells, corresponding to low-positive expression (1–49% tumor cell staining).

### 2.5. Therapeutic Intervention

Because of the confirmed nodal involvement, the case was discussed by the institutional multidisciplinary tumor board, which recommended adjuvant systemic therapy. The patient subsequently received four cycles of cisplatin (75 mg/m^2^ on day 1) plus pemetrexed (500 mg/m^2^ on day 1), administered every 21 days. Folic acid and vitamin B12 supplementation were provided according to institutional protocols, along with standard antiemetic prophylaxis. She completed all four cycles without dose reduction, treatment delay, or premature discontinuation, and the treatment was well tolerated. Given the EGFR L858R mutation and the evidence from the ADAURA trial supporting adjuvant osimertinib in completely resected stage IB–IIIA EGFR-mutated NSCLC, osimertinib was started at 80 mg once daily approximately five months after surgery, with a planned duration of three years.

### 2.6. Follow-Up and Outcomes

**Figure 3:**
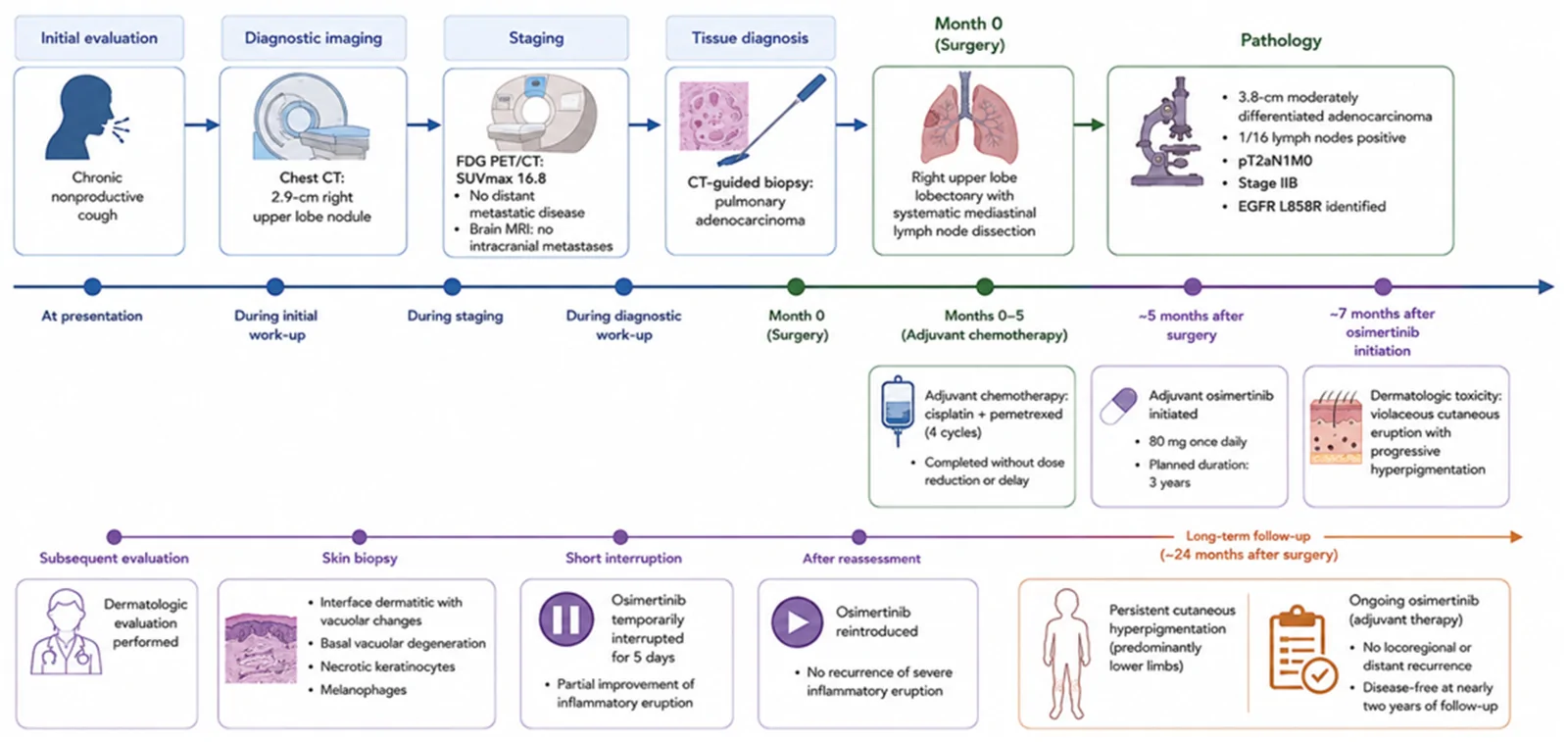
Clinical Timeline from Diagnosis to Ongoing Adjuvant Osimertinib. The timeline presents only clinically relevant events, including diagnostic evaluation, right upper lobectomy with systematic lymph node dissection, pathological staging as pT2aN1M0 (stage IIB) with EGFR L858R identification, adjuvant cisplatin plus pemetrexed, initiation of Osimertinib, onset and management of violaceous rash and cutaneous hyperpigmentation, temporary treatment interruption for five days, treatment reintroduction, and ongoing follow-up without evidence of disease recurrence. Non-clinical manuscript-related items were removed. Created with BioRender.com.

During follow-up (Figure 3), the patient remained under close multidisciplinary surveillance involving oncology, dermatology, nutrition, and psychological support services. Early adverse events during Osimertinib therapy included grade 1 diarrhea, transient anorexia associated with mild weight loss, and self-limited arthralgia.

### 2.7. Dermatologic Toxicity

**Figure 4:**
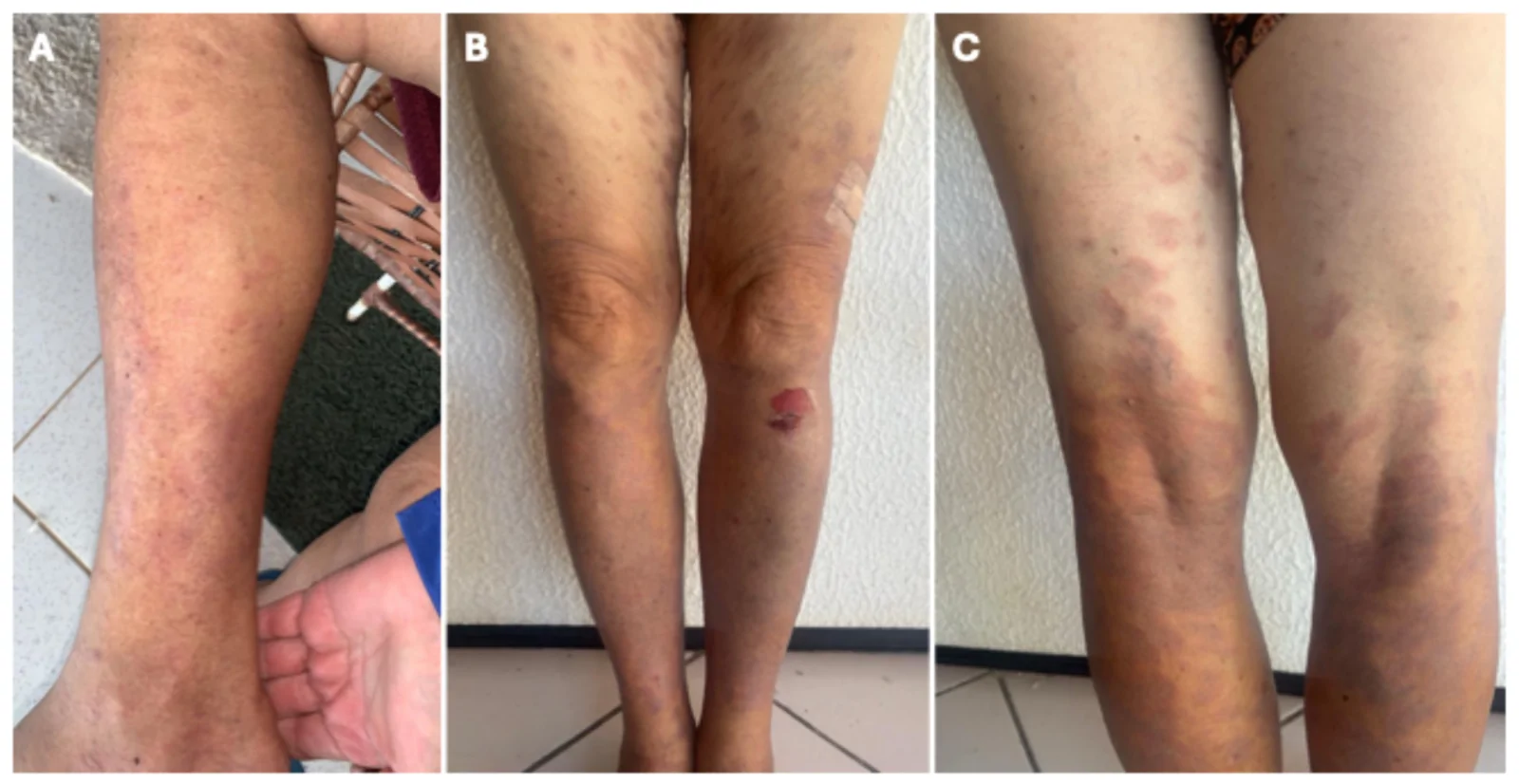
Clinical evolution of the cutaneous toxicity associated with adjuvant Osimertinib therapy. (**A**) Initial onset of violaceous macules and patches involving the upper and lower extremities. (**B**) Progressive development of symmetric violaceous-brown hyperpigmented macules and confluent patches predominantly involving both lower limbs after partial regression of the inflammatory eruption. (**C**) Close-up of the persistent mottled violaceous-brown hyperpigmentation of the lower leg, compatible with post-inflammatory pigmentary changes.

Approximately seven months after initiating adjuvant osimertinib, the patient developed an unusual and progressive violaceous cutaneous eruption, initially affecting both upper and lower extremities (Figure 4A). The lesions were accompanied by progressive skin darkening and were clinically classified as grade 2 according to the Common Terminology Criteria for Adverse Events (CTCAE v5.0). The eruption was clinically characterized by violaceous macules and patches. No quantitative assessment of affected body surface area was documented at the time of the event. No ulceration, blistering, secondary infection, mucosal involvement, or significant pruritus was observed. Over subsequent months, the inflammatory eruption evolved into diffuse cutaneous hyperpigmentation, particularly affecting the lower limbs (Figure 4B,C). The upper-extremity lesions became less prominent, while the residual hyperpigmentation persisted predominantly in the lower limbs.

Although there were no features of severe cutaneous toxicity or functional impairment, the progressive and persistent nature of the eruption prompted a short therapeutic and diagnostic interruption of osimertinib. The treatment was withheld for five days to assess whether the inflammatory component would improve with drug withdrawal and to facilitate dermatologic evaluation and exclusion of alternative causes. Partial improvement of the violaceous eruption was observed during this interval, supporting the subsequent decision to cautiously reintroduce osimertinib after multidisciplinary reassessment.

**Figure 5:**
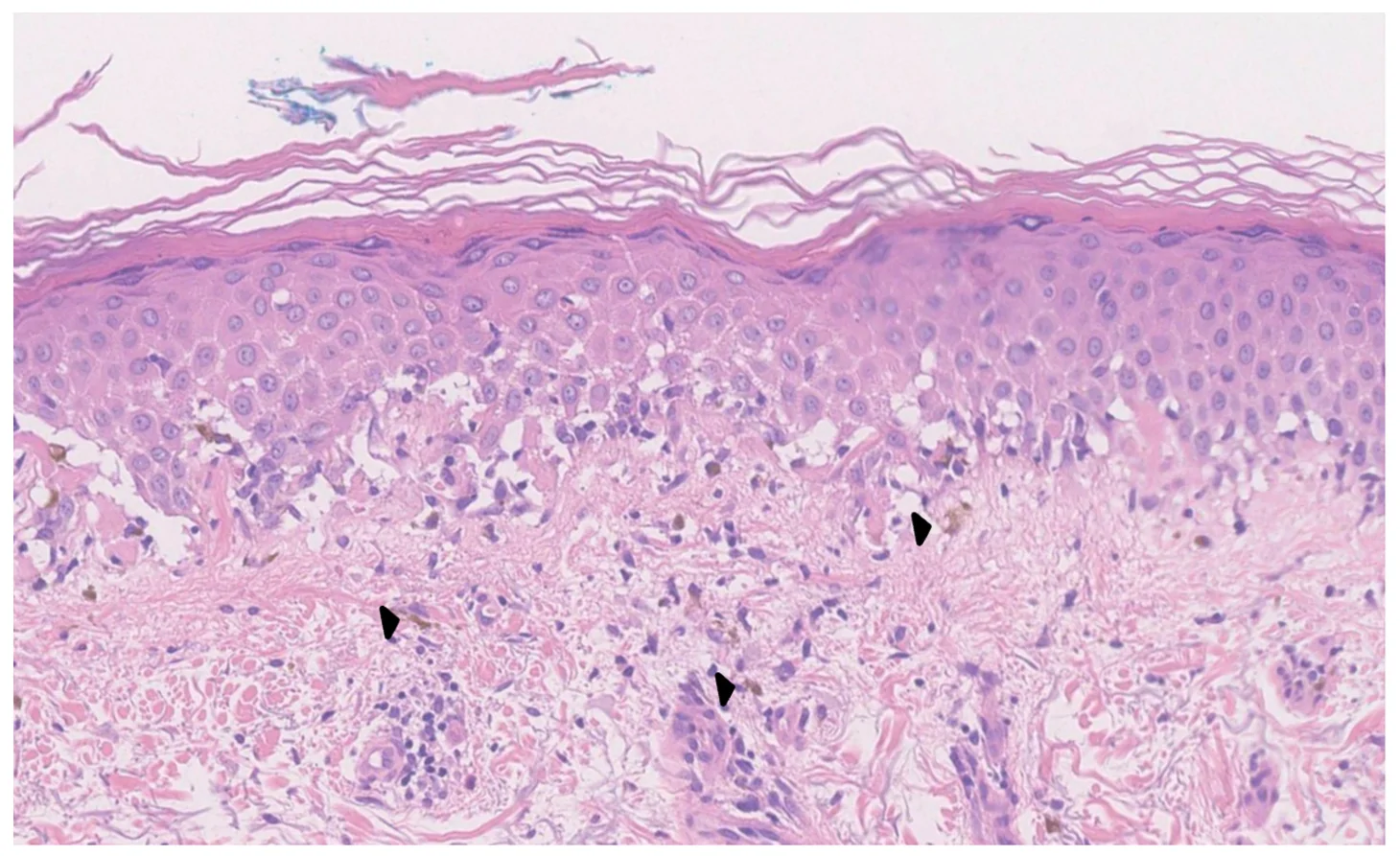
Histopathologic findings of Osimertinib-associated interface vacuolar dermatitis showing interface dermatitis with vacuolar changes. There is moderate-to-marked basal vacuolar degeneration with frequent necrotic keratinocytes at the dermoepidermal junction, mild spongiosis, and a sparse superficial perivascular lymphocytic infiltrate. Melanophages are noted in the papillary dermis (arrows) (H&E × 20).

A skin biopsy of the right thigh was subsequently performed to further investigate the persistent cutaneous manifestations. Histopathological examination demonstrated interface dermatitis with vacuolar changes characterized by moderate to marked basal vacuolar degeneration, mild spongiosis, frequent necrotic keratinocytes at the base of the epidermis, and a mild superficial lymphocytic inflammatory infiltrate accompanied by melanophages (Figure 5). The clinicopathological diagnosis was an interface dermatitis with vacuolar degeneration and subsequent post-inflammatory hyperpigmentation, considered compatible with a drug-related cutaneous eruption. The main differential diagnoses included lichenoid drug eruption, erythema multiforme, fixed drug eruption, drug-induced pigmentation, and post-inflammatory hyperpigmentation secondary to the preceding inflammatory eruption. Pemetrexed and cisplatin were considered relevant alternative drug exposures because both had been administered before the onset of the cutaneous manifestations and have been associated with pigmentary changes.

Chronic concomitant medications, including losartan, lercanidipine, evogliptin, and levothyroxine, were also considered as potential confounding factors. The clinical distribution, absence of mucosal involvement, absence of blistering or ulceration, and the subsequent evolution of the lesions were considered in the differential diagnosis. No additional autoimmune or serologic work-up was performed specifically for the cutaneous eruption; this is acknowledged as a limitation of the diagnostic assessment. Here, the findings were considered most consistent with a drug-associated interface reaction followed by persistent post-inflammatory hyperpigmentation, although a specific offending agent could not be established with certainty.

Pemetrexed and cisplatin have both been reported to cause cutaneous pigmentation. However, the temporal relationship favored Osimertinib as the most likely culprit: the violaceous eruption developed approximately seven months after initiation of Osimertinib, progressed during continued exposure, and partially improved following temporary treatment interruption. In addition, the inflammatory component did not recur after Osimertinib was subsequently reintroduced. The biopsy findings provided objective clinicopathological support for a drug-related interface reaction, although they were not specific for Osimertinib.

A Naranjo Adverse Drug Reaction Probability Scale assessment yielded a score of 3, corresponding to a possible drug-related adverse reaction. The temporal association was considered supportive, as the violaceous eruption developed approximately seven months after initiation of osimertinib and progressed during continued exposure. Histopathological examination demonstrated an interface vacuolar dermatitis compatible with a drug-induced reaction but not specific for osimertinib. Partial improvement of the inflammatory eruption following temporary treatment interruption, followed by recurrence after treatment reintroduction, provided supportive evidence from the rechallenge. However, the absence of recurrence of the inflammatory eruption after osimertinib rechallenge limited the strength of the causal inference. Plausible alternative causes, particularly previous exposure to pemetrexed and cisplatin and concomitant medications, were also considered. Overall, the findings support a possible association with osimertinib, but do not establish a definitive causal relationship.

The lesions remained confined to the extremities, without facial or mucosal involvement. The patient was referred for dermatologic evaluation, and desloratadine, a second-generation, nonsedating H1-antihistamine, was initiated as symptomatic treatment for the inflammatory cutaneous eruption, given the potential contribution of histamine-mediated mechanisms to EGFR inhibitor-associated cutaneous symptoms [14]. Treatment with desloratadine resulted in partial resolution of the inflammatory eruption, with regression of the violaceous rash. However, cutaneous hyperpigmentation persisted despite improvement of the inflammatory component. Owing to persistent dermatologic toxicity, Osimertinib was temporarily interrupted for five days and subsequently resumed after multidisciplinary reassessment, without recurrence of severe cutaneous symptoms.

**Figure 6:**
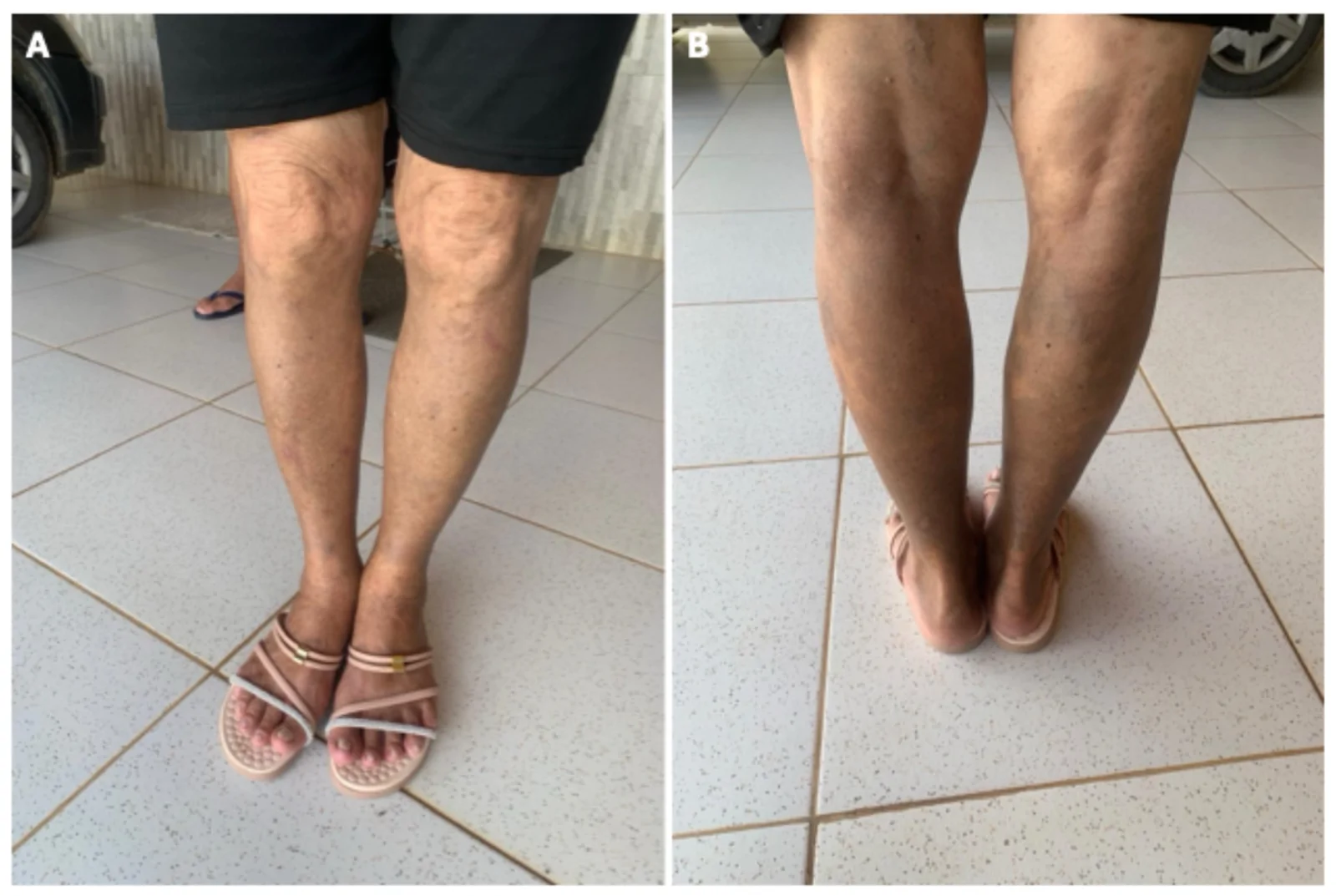
Current clinical appearance after nearly two years of follow-up. (**A**) Anterior and (**B**) posterior views of the lower extremities showing sustained improvement of the inflammatory violaceous eruption during daily desloratadine therapy, with persistent bilateral diffuse violaceous-brown post-inflammatory hyperpigmentation during continued adjuvant Osimertinib treatment.

During follow-up, residual hyperchromic discoloration persisted predominantly in the lower extremities, although the violaceous eruption gradually improved. Serial imaging demonstrated no locoregional recurrence or distant metastatic disease. At the latest follow-up, the patient remained disease-free, maintained an ECOG performance status of 0, and continued adjuvant Osimertinib with daily desloratadine. Persistent violaceous discoloration and diffuse cutaneous hyperpigmentation remained clinically stable, particularly in the lower limbs, without pruritus, ulceration, secondary infection, or functional impairment (Figure 6). The patient reported no interference with daily activities.

## 3. Discussion

The present case illustrates an uncommon and prolonged dermatologic toxicity occurring during adjuvant osimertinib therapy in a patient with resected stage IIB (pT2aN1M0) EGFR L858R-mutated lung adenocarcinoma. Following surgical resection and adjuvant platinum-based chemotherapy, osimertinib was initiated according to the planned adjuvant treatment strategy. At the latest follow-up, the patient remained without clinical or radiological evidence of disease recurrence and continued osimertinib, while the cutaneous hyperpigmentation remained clinically stable.

Osimertinib is a third-generation EGFR tyrosine kinase inhibitor selectively targeting sensitizing EGFR mutations and the T790M resistance mutation while exhibiting reduced activity against wild-type EGFR [15]. This increased selectivity is believed to contribute to its more favorable toxicity profile compared with earlier-generation EGFR inhibitors [15,16]. In the ADAURA [9], AURA [17], and FLAURA [18] trials, the most frequently reported dermatologic adverse events included acneiform rash, xerosis, pruritus, and paronychia, which were predominantly low-grade and manageable with supportive measures. Severe dermatologic toxicity was uncommon, and treatment discontinuation due to skin-related adverse events was rare.

The dermatologic manifestations observed in the present case differed substantially from the classical toxicity profile reported in clinical trials [9,17,18]. Rather than a transient acneiform eruption, the patient developed a persistent violaceous eruption that subsequently evolved into long-lasting cutaneous hyperpigmentation. The pigmentary alterations remained evident for more than one year after their initial appearance and persisted despite temporary treatment interruption and improvement of the inflammatory component. This prolonged clinical evolution, together with the biopsy demonstrating interface vacuolar dermatitis with basal vacuolar degeneration, necrotic keratinocytes, and melanophages, represents the main distinctive feature of the present case.

Previous reports have described uncommon pigmentary reactions during osimertinib therapy, including ashy dermatosis-like hyperpigmentation [19,20]. These reports are particularly relevant to the present case because they involve persistent pigmentary changes during osimertinib exposure. However, the clinical and histopathological phenotype observed in our patient differs from these previously reported cases, with an initial violaceous eruption followed by persistent post-inflammatory hyperpigmentation and biopsy-confirmed interface dermatitis. Other rare cutaneous toxicities associated with osimertinib, such as vasculitis and toxic epidermal necrolysis, have also been reported [19,21,22], but these conditions were not considered directly comparable to the present pigmentary/interface phenotype and were therefore excluded from the focused comparison in Table 2. In contrast, the present case demonstrates a sequential clinicopathological phenotype characterized by violaceous interface dermatitis followed by persistent post-inflammatory hyperpigmentation, with continued Osimertinib exposure after temporary interruption (Table 2).

Our findings are particularly noteworthy when considered in the context of the dermatologic safety profile previously reported for Osimertinib. In a comprehensive review by Chu et al. [23], Osimertinib was associated with a lower frequency and severity of dermatologic adverse events compared with first- and second-generation EGFR tyrosine kinase inhibitors, with most cases consisting of mild acneiform rash, xerosis, pruritus, and paronychia that were generally manageable with supportive care. The study emphasized that the dermatologic toxicity profile of Osimertinib appears distinct from earlier EGFR-TKIs and rarely necessitates treatment discontinuation. In contrast, our patient developed an unusual violaceous rash followed by persistent cutaneous hyperpigmentation, a presentation not commonly described in the literature. An additional interesting aspect of this case was the coexistence of a rare CD274 (PD-L1) exon 7 rearrangement and low-level PD-L1 expression (20% of tumor cells). Although the biological implications of PD-L1 rearrangements in EGFR-mutated NSCLC remain incompletely understood, these alterations have been associated with dysregulated immune checkpoint signaling and may contribute to tumor immune escape [24]. The concomitant presence of an activating EGFR mutation, low tumor mutational burden (4 mutations/Mb), microsatellite stability, and PD-L1 pathway alteration highlights the molecular heterogeneity that can coexist within apparently typical EGFR-driven tumors [25,26].

An additional strength of the present report is the histopathologic confirmation of drug-induced skin toxicity. Skin biopsy demonstrated interface vacuolar dermatitis with basal vacuolar degeneration, necrotic keratinocytes, and melanophages, a pattern consistent with drug-induced dermatosis and reminiscent of erythema multiforme. These findings provide objective clinicopathological evidence supporting a drug-induced interface reaction, although they are not specific for Osimertinib. Given the prior exposure to pemetrexed and cisplatin, both of which have been associated with cutaneous pigmentation, these agents must be considered in the differential diagnosis. However, the clinical chronology favored Osimertinib because the eruption developed approximately seven months after Osimertinib initiation, progressed during ongoing exposure, and partially improved after temporary treatment interruption. The absence of recurrence of the inflammatory eruption after Osimertinib reintroduction further suggests that the persistent pigmentation may represent a post-inflammatory sequela rather than ongoing active drug hypersensitivity.

Moreover, pigmentary alterations remained evident despite improvement of the inflammatory component after treatment with desloratadine and temporary Osimertinib interruption. This observation expands the spectrum of recognized Osimertinib-associated dermatologic toxicities and highlights the need for greater awareness of atypical and potentially long-lasting pigmentary manifestations during prolonged treatment exposure.

Although pigmentary disorders have occasionally been reported with EGFR-targeted therapies (Table 2), their pathophysiology remains incompletely understood. As summarized in Table 2, most published reports of Osimertinib-associated dermatologic toxicity describe either severe hypersensitivity reactions such as toxic epidermal necrolysis or isolated pigmentary disorders resembling ashy dermatosis [19,20,21,22,27,28]. Other rare dermatologic toxicities, including vasculitis and toxic epidermal necrolysis, have also been reported during osimertinib therapy, but these entities were not included in the focused comparison because they represent distinct inflammatory or severe cutaneous adverse reaction phenotypes rather than pigmentary or interface/lichenoid reactions. In contrast, our patient presented a distinct clinical phenotype characterized by a persistent violaceous eruption followed by long-lasting hyperpigmentation, occurring during adjuvant Osimertinib therapy and successfully managed without permanent treatment discontinuation.

**Table 2:** Reported cases of rare Osimertinib-associated dermatologic toxicities and comparison with the present case.

**Ref.**	**Patients**	Dermatologic Manifestation	Time to Onset	Management	Outcome
Present case	71-year-old woman with resected stage IIB EGFR L858R-mutated lung adenocarcinoma receiving adjuvant Osimertinib	Persistent violaceous rash followed by diffuse cutaneous hyperpigmentation, predominantly affecting upper and lower extremities	~7 months	Desloratadine, temporary Osimertinib interruption, dermatologic follow-up, treatment reintroduction	Rash improved; persistent lower-limb hyperpigmentation remained; Osimertinib was subsequently continued; no disease recurrence was observed during the available follow-up
[21]	49-year-old woman with EGFR-mutated NSCLC	Cutaneous vasculitis presenting as palpable purpura of the lower extremities	11 days	Osimertinib discontinuation, methylprednisolone, cetirizine	Complete resolution after drug withdrawal
[19]	44-year-old woman with EGFR-mutated lung adenocarcinoma	Toxic epidermal necrolysis (TEN) with diffuse erythematous rash, vesicles, purpuric macules, and severe mucositis	10 days	Systemic corticosteroids, immunoglobulin, supportive care	Remarkable recovery
[20]	Patient with EGFR-mutated NSCLC	Ashy dermatosis-like hyperpigmentation	Not reported	Osimertinib dose reduced to 40 mg/day	Hyperpigmentation persisted despite dose reduction; treatment continued
[27]	71-year-old woman with EGFR L858R-mutated NSCLC	Generalized ashy dermatosis-like slate-gray hyperpigmentation involving trunk and extremities	~6 months	Osimertinib continued; no specific intervention reported	Hyperpigmentation remained stable; treatment maintained
[22]	80-year-old woman with EGFR-mutated lung adenocarcinoma	Toxic epidermal necrolysis (TEN) with diffuse erythema, epidermal detachment, and blistering	46 days	Osimertinib discontinuation, corticosteroid, intravenous immunoglobulin	Clinical recovery after intensive treatment
[28]	Patients receiving EGFR inhibitors, including Osimertinib	Painful cut-like lesions affecting fingertips and lateral fingers	~4 weeks	Topical treatment or drug discontinuation	Partial or complete resolution

In the previous cases, rare dermatologic toxicities occurred across different disease settings, including both resected/adjuvant and advanced/metastatic disease. In some metastatic cases, clinical or radiological tumor responses were documented despite the occurrence of dermatologic toxicity; however, the limited number of cases, heterogeneous disease settings, and incomplete reporting of oncologic outcomes preclude any firm inference regarding a relationship between these rare adverse events and treatment efficacy.

Several mechanisms may contribute to these manifestations, including chronic EGFR blockade-induced epidermal inflammation, post-inflammatory melanocyte activation, dysregulated keratinocyte–melanocyte signaling, and drug-induced alterations in melanin synthesis and distribution [21]. In the present case, the persistence of hyperchromic lesions long after the initial inflammatory phase suggests that post-inflammatory pigmentary remodeling may have played an important role. An additional clinically relevant aspect of this case is the management of dermatologic toxicity without permanent treatment discontinuation. Here, temporary interruption of osimertinib, combined with dermatologic evaluation and close follow-up, allowed symptom stabilization and subsequent treatment reintroduction. Osimertinib was subsequently continued without recurrence of severe cutaneous symptoms.

As the use of adjuvant osimertinib continues to expand worldwide, clinicians will increasingly encounter patients receiving prolonged exposure to EGFR-targeted therapy [10,29,30,31]. Long-term data from the ADAURA trial and its translational analyses have further characterized patterns of recurrence and molecular residual disease during and after adjuvant treatment. In a recent molecular residual disease (MRD) analysis of the ADAURA trial, Herbst et al. [31] reported that many MRD or disease-free survival events occurred after completion or discontinuation of adjuvant osimertinib.

Circulating tumor DNA-based MRD detection also preceded radiologic recurrence in a substantial proportion of cases, providing insight into patterns of residual disease during follow-up [31]. These findings provide important context for the planned duration of adjuvant therapy but do not allow conclusions regarding the oncologic effect of treatment interruption or continuation in an individual patient. In the present case, osimertinib was reintroduced after a five-day interruption following multidisciplinary reassessment, and no disease recurrence was observed during the available follow-up. However, this clinical course should not be interpreted as evidence of an individual oncologic benefit attributable to treatment continuation.

From a patient-centered perspective, the persistent skin discoloration did not result in functional impairment or lead to treatment discontinuation. The patient remained willing to continue adjuvant osimertinib and considered the multidisciplinary follow-up and supportive care important for maintaining treatment adherence. This aspect is clinically relevant because visible and persistent cutaneous changes may represent a concern for patients even when they are not associated with pain, pruritus, or functional limitation. In this case, continued communication between oncology and dermatology facilitated management of the inflammatory component while allowing ongoing cancer-directed therapy.

Recognition of atypical dermatologic manifestations, including persistent pigmentary alterations, is essential to avoid unnecessary treatment discontinuation and to facilitate timely referral to dermatology specialists. Further reports are needed to better characterize the incidence, biological mechanisms, and optimal management of these uncommon toxicities.

## 4. Patient Perspective

The patient expressed satisfaction with the treatment strategy and reported confidence in continuing long-term Osimertinib despite manageable adverse effects. She considered the multidisciplinary follow-up and supportive care essential for maintaining treatment adherence.

## 5. Conclusions

This case describes a rare and persistent dermatologic manifestation temporally associated with adjuvant Osimertinib, characterized by violaceous rash and long-lasting cutaneous hyperpigmentation in a patient with completely resected stage IIB EGFR L858R-mutated lung adenocarcinoma. Despite requiring temporary treatment interruption, the inflammatory cutaneous manifestations improved sufficiently to allow Osimertinib reintroduction and continuation. At the latest follow-up, the patient had no clinical or radiological evidence of disease recurrence, while the residual hyperpigmentation remained clinically stable. Increased awareness of atypical cutaneous adverse events may facilitate appropriate dermatologic assessment and multidisciplinary management during long-term EGFR-targeted therapy.

## Figures and Tables

**Table 1:** Comprehensive molecular characterization of the tumor.

Category	Platform	Finding
Specimen characteristics	ARGEN 50 NGS panel	60% viable tumor in the analyzed block
Sequencing quality	ARGEN 50 NGS panel	1,050,512 mapped DNA reads and 199,551 mapped RNA reads; 99.40% base-coverage uniformity; 100% of bases covered at ≥350× and ≥500×
Initial molecular profiling	ARGEN 50 NGS panel	EGFR L858R (VAF 9.72%); no reportable alterations detected in ALK, BRAF, ERBB2, KRAS, MET, RET, ROS1, or NTRK1/2/3
Driver alteration	FoundationOne CDx	EGFR exon 21 L858R (VAF 18.1%)
Immune-related alteration	FoundationOne CDx	CD274 (PD-L1) exon 7 rearrangement
Tumor suppressor alterations	FoundationOne CDx	APC Q1549 * (VAF 13.7%); CDKN2A loss; CDKN2B loss; RBM10 E380 * (VAF 6.8%)
Metabolic alteration	FoundationOne CDx	MTAP loss
Wnt pathway alteration	FoundationOne CDx	RNF43 S607L (VAF 5.3%)
Possible clonal hematopoiesis-related variant	FoundationOne CDx	DNMT3A S770L, subclonal (VAF 1.3%)
Microsatellite status	FoundationOne CDx	Microsatellite stable (MSS)
Tumor mutational burden	FoundationOne CDx	4 mutations/Mb
Sequencing coverage	FoundationOne CDx	Median exon coverage: 2264×
Negative findings	FoundationOne CDx	No reportable alterations detected in ALK, BRAF, ERBB2, KRAS, MET, RET, or ROS1

* Indicates a stop-gain (nonsense) variant.

## Data Availability

The original contributions presented in this study are included in the article. Further inquiries can be directed to the corresponding author.

## Abbreviations

The following abbreviations are used in this manuscript:

AJCC	American Joint Committee on Cancer
CT	Computed tomography
CTCAE	Common Terminology Criteria for Adverse Events
EGFR	Epidermal growth factor receptor
ECOG	Eastern Cooperative Oncology Group
FDG	Fluorodeoxyglucose
H&E	Hematoxylin and eosin
mMRC	Modified Medical Research Council
MIP	Maximum intensity projection
MRD	Molecular residual disease
MSS	Microsatellite stable
NGS	Next-generation sequencing
NSCLC	Non-small cell lung cancer
PD-L1	Programmed death-ligand 1
PET	Positron emission tomography
STAS	Spread through air spaces
TKI	Tyrosine kinase inhibitor
TMB	Tumor mutational burden
VAF	Variant allele frequency
